# Identifying innovative models of urgent care in rural coastal areas in England: the Elevate study - a mixed-methods protocol

**DOI:** 10.1136/bmjopen-2026-116557

**Published:** 2026-02-24

**Authors:** Pete Lampard, Joy Adamson, Helen Anderson, Lisa Ballantine, Fiona Bell, Jonathan Richard Benger, Rebecca Louise Blakey, Phillip Dickinson, Steven Dykes, James Gaughan, Stuart Maitland-Knibb, Daniel Mensah, Zacharia Alice Ransome, Gerry Richardson, Rita Santos, Rebecca Sheridan, Peter Sivey, Ed Smith, Wei Song, Martin Sutcliffe, Cameron Jack Stockwell, Sarah Voss, Arabella Scantlebury

**Affiliations:** 1 York Trials Unit, Department of Health Sciences, University of York, York, UK; 2 Research and Innovation, York and Scarborough Teaching Hospitals NHS Foundation Trust, York, UK; 3 Yorkshire Ambulance Service NHS Trust, Wakefield, Wakefield, UK; 4 Academic Department of Emergency Care, The University Hospitals NHS Foundation Trust, Bristol, UK; 5 Faculty of Health & Life Sciences, The University of the West of England, Bristol, UK; 6 Age UK Scarborough, Scarborough, UK; 7 SeeCHANGE Scarborough, Scarborough, UK; 8 East Midlands Ambulance Service NHS Trust, Nottingham, UK; 9 Centre for Health Economics, University of York, York, UK; 10 School of Medicine and Dentistry, University of Lancashire, Preston, UK; 11 Centre for Evidence and Implementation Science, University of Birmingham, Birmingham, UK; 12 Centre of Health Economics, University of York, York, UK; 13 York and Scarborough Teaching Hospitals NHS Foundation Trust, York, UK

**Keywords:** accident & emergency medicine, emergency service, hospital, frail elderly, health policy, health services accessibility

## Abstract

**Abstract:**

**Introduction:**

Urgent and emergency care (UEC) systems in England face unprecedented pressures, with record accident and emergency attendances, persistent breaches of ambulance response targets and poorer outcomes for time-sensitive conditions. National UEC recovery plans have introduced multiple innovations—such as same-day emergency care, virtual wards and specialty hubs—to manage these pressures and improve patient flow. Rural coastal areas are particularly vulnerable to excessive demand due to higher levels of deprivation, older populations with complex health needs, seasonal surges that generate unpredictable demand and challenges in attracting and retaining staff. Following the Chief Medical Officer’s 2021 Annual Report, funding research and developing bespoke solutions to manage UEC demand and address geographical disparities has been recognised as a national priority. The Elevate study responds to this priority by identifying and evaluating innovative models of UEC in rural coastal communities in England.

**Methods and analysis:**

The Elevate study is a 30-month, mixed-methods evaluation that comprises three interlinked work packages: (1) *National service mapping*—outlining provision of innovative models of UEC in rural coastal areas of England. This will be developed through document review and interviews with regional and national service leaders. (2) *Quantitative analysis*—quasiexperimental and longitudinal approaches will use National Health Service (NHS) England’s Emergency Care Data Set and linked routine NHS datasets to evaluate the impact of UEC models on health and process outcomes. Standard and bespoke metrics will be developed and used to assess performance. (3) *Qualitative case studies*—up to 12 case studies of UEC models in rural coastal communities. Interviews with patients and staff and non-participant observation will explore how and why different UEC models influence patient experience, clinical outcomes, resource use and the workforce. Findings will be integrated using the Consolidated Framework for Implementation Research to identify components of UEC models that are effective, scalable and sensitive to local context,

**Ethics and dissemination:**

Ethical approval for qualitative components was granted by the North of Scotland Research Ethics Committee (25/NS/0099). Dissemination will include peer-reviewed publications, policy briefs, creative media and community engagement activities to ensure findings are communicated inclusively and effectively to policymakers, health and social care practitioners and the public.

**Trial registration number:**

Research Registry (researchregistry11126).

**STRENGTHS AND LIMITATIONS OF THIS STUDY**

- The mixed-methods design integrates quantitative and qualitative approaches to provide a comprehensive evaluation of urgent and emergency care models.
- Use of national routine datasets enables large-scale analysis of emergency care performance across rural coastal areas.
- Linking multiple data sources (Emergency Care Data Set, Hospital Episode Statistics Admitted Patient Care, and local ambulance data) allows a detailed assessment of patient pathways and outcomes.
- Qualitative case studies offer in-depth insights into contextual factors influencing implementation and effectiveness.
- Reliance on routine datasets may limit completeness and timeliness of quantitative analyses.

## Introduction

 International evidence indicates mounting pressures on urgent and emergency care (UEC) services.^1^ In England, record high total accident and emergency (A&E) attendances were reported in the quarter April–June 2025^2^ and ambulance response time targets have been consistently missed across all categories of acuity since early 2021.^3^

In 2023, NHS England (NHSE) outlined a 2-year delivery plan to recover UEC services.^4^ This plan has called for the introduction of numerous demand-based initiatives, promoting new ways to organise and deliver care to manage pressure on UEC. These include: same-day emergency care (SDEC),^5^ virtual wards^6^ and specialty-specific services such as acute respiratory infection (ARI) hubs.^7^ Common to these initiatives is the desire to reduce UEC demand by shifting care, where appropriate, from the hospital to the community, by speeding up discharge from hospitals and by connecting patients to the right care as early as possible in their patient journey.^4^ This emphasis aligns with long-standing policy directives from consecutive governments, which have consistently prioritised the integration of care and the strengthening of community-based services—a direction reaffirmed in the NHS Long Term Plan.^8^

Pressure on UEC represents a *whole system issue*. However, rural coastal areas are particularly vulnerable to excessive demand due to, for example, higher levels of deprivation, a high burden of mental ill-health and an older age profile with a higher risk of multiple long-term conditions.^9^,^14^ They also face unpredictable demand due to seasonal variation, with larger populations due to seasonal tourism alongside winter pressures; difficulties attracting and retaining staff; and sparsity of services leading to increased travel times and more difficult access.^15^,^17^ The pressure on UEC in rural coastal communities has captured attention across authorities and public bodies, with successive governments framing the need to prioritise the health and well-being of communities in these areas as a key concern, and the latest 10-year plan explicitly highlighting it as a priority.^8 16 17^

Rural coastal areas are under-researched, as well as underserved. According to the England Chief Medical Officer’s 2021 annual report, ‘coastal communities have long been overlooked with limited research on their health and well-being’.^17^ This is compounded by evaluations of national policy typically privileging a ‘one-size-fits-all’ approach over the development of locality-specific evidence.^18^,^20^ While evidence relating to the impact of innovative service delivery in UEC is growing,^21^,^26^ evidence on the specific impacts of innovations in rural coastal settings remains limited (excepting emerging work on community first responders and workforce recruitment and retention).^27 28^

With the recent launch of the UEC recovery programme as a backdrop,^4^ our study—in response to a commissioned call from the National Institute for Health and Care Research^29^—aims to identify and evaluate innovative models of UEC in rural coastal communities in England; that is, new models of care at the service or organisational level aiming to improve UEC provision in rural coastal areas. Evaluation includes a model’s impact on patient care, clinical outcomes, workforce and use of healthcare resources. Aligning with the Chief Medical Officer’s call for evidence-informed, locally tailored strategies, this includes generating insights that inform bespoke service design for rural coastal contexts. Our findings will aim to shape commissioning priorities, workforce planning and clinical care models, ensuring they reflect the unique challenges and opportunities of these communities.

A summary of the Elevate study’s specific objectives is provided in table 1, along with the work packages (WPs) designed to achieve them.

**Table 1:** Research objectives and work packages

Research objectives	Work packages
To map the models of UEC that exist in rural coastal England.	1
To explore how and why new UEC models in rural coastal areas impact clinical outcomes and the wider healthcare system, as well as their resource use.	2, 3
To explore how and why new UEC models in rural coastal areas impact patient and/or care experience and the UEC workforce.	3
To determine the implications of new UEC models in rural coastal areas for health inequalities (eg, access to healthcare for underserved groups).	1, 2, 3
To assess which models and/or components of UEC within rural coastal communities should be scaled up and implemented to improve service provision within rural coastal communities.	1, 2, 3

UEC, urgent and emergency care.

## Methods and analysis

### Study design

**Figure 1:**
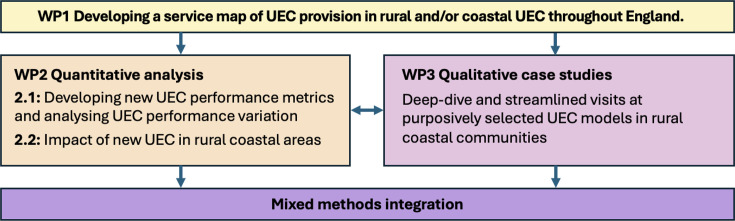
Study overview: three integrated work packages of the Elevate study. UEC, urgent and emergency care; WP, work packages.

This is a mixed-methods study comprised of three interlinking WPs (figure 1). Our study design is based on the methodology we have successfully applied to a number of other, recently completed or nearing completion, NIHR-funded, national mixed-methods evaluations of acute and emergency care directives.^20 30 31^ All three WPs are designed to continually inform each other, while enabling us to be responsive to the changing policy landscape within the NHS in England.

For this project, we have convened an ‘expert panel’, including relevant coapplicants, external stakeholders (from general practice, social care, ambulance services, emergency medicine, operational managers, integrated care boards, the National Centre of Remote and Rural Medicine, community organisations and academics) and community representatives from rural coastal communities. Membership of this panel will be iterative and responsive to findings across WPs. The panel will be convened at strategic points throughout the project (eg, when selecting case sites for WP 3) but will act as a ‘pool of experts’ we can draw on for ad hoc, service specific advice when needed.

#### WP 1: Develop a ‘service map’ of UEC innovation in rural coastal UEC services throughout England (months 1–14)

##### Aim

While the UEC recovery plan has empowered services to innovate, as well as showcasing potential models of UEC provision, it does not advocate for any particular model of care, remaining open to local adaptation. This complicates any national evaluation, given the potential multiplicity of interventions. The aim of WP 1 is to identify innovative models of care introduced in rural coastal areas of England in response to the UEC recovery plan.

##### Design

Working with our expert panel and community engagement and involvement (CEI) communities, we will coproduce a working definition of rural coastal that reflects the identities of both local services and populations.

We will develop a comprehensive and descriptive national map of models of UEC provision across rural coastal areas in England, including active services introduced from 2016 onwards and those planned during the study period, aligning with recent national policy and service transformation phases. This will include the *location* of innovative models of UEC, the *dates* that these models were or will be introduced and details regarding the *model of care* (including the services involved, priorities being addressed and workforce features).

The service map will be informed by a rapid desktop review of key policy and service documents relevant to rural coastal areas. This will include UEC policies, strategies and plans at local, regional and national (for example, NHSE) levels, providing contextual intelligence on rural coastal health systems and identifying UEC service models relevant to the study. Iterative guidance and support for the service map’s development will be supplied through interviews with two groups of key informants. The first group will include national and regional UEC organisations (such as the Department for Health and Social Care, NHSE and the Royal College of Emergency Medicine), to build an understanding of national UEC directives and if/how they have been interpreted and applied to rural coastal areas, as well as future plans for UEC and its application in rural coastal areas. The second will consist of service leaders from rural coastal sites identified as areas of interest, to obtain detailed information on UEC service models in rural coastal areas, as well as the drivers that underpin innovation, local tailoring to national directives, challenges to improving UEC and how these challenges might be addressed.

##### Outputs

The service map will provide up-to-date information about UEC models and the types of organisational-level innovations that exist. This will provide the data required for qualitative evaluation in WP 3 (including potential case sites and gatekeepers) and our quantitative analyses in WP 2. The service map will form the basis of peer-to-peer and cross-agency learning, both regionally and nationally, which we will use to create a peer network for more immediate and sustained impact.

### WP 2: Quantitative analysis of innovative UEC models in rural coastal communities (months 1–27)

#### Aim

To assess the impact of different UEC models through standard performance indicators and the development of new UEC performance metrics.

#### Design

We will primarily use pseudonymised Emergency Care Data Set (ECDS) provided by NHSE,^32^ a comprehensive national dataset with information for all attendances to English A&E departments, from which we will capture patient demographics and attendance acuity, diagnosis and timestamps (eg, arrival and departure time) for all English A&E attendances. Using patient pseudonymised IDs, we will link this to the Hospital Episode Statistics Admitted Patient Care (HES APC) dataset to quantify patients comorbidities from prior admissions. We will integrate this with local ambulance data where available.

Using the ECDS and HES APC datasets, we will develop new UEC performance metrics to analyse performance variation across England. The ECDS includes a rich set of information on all attendances to UEC, such as chief complaint, acuity, diagnosis, treatments, investigations, time and mode of arrival as well as time and destination of discharge, along with patient characteristics, including age, sex and local area of residence. The patient’s local area of residence allows linkage with ONS local area information, such as rurality and socioeconomic deprivation. The HES APC will enable a link to previous hospital admissions and diagnoses, allowing us to capture patients’ health status. Combining these, we will calculate several standard metrics of health and process outcomes for emergency care.

Metrics will include emergency admission rates, non-urgent attendances (for type 1 EDs),^33^ waiting time to triage, waiting time to discharge—including admission, frequent attenders, attendances arriving by ambulance, attendances by referral source (eg, GP, by other hospital service), reattendance, ‘did not wait’ attendances, costs of attendance and emergency admissions to understand the variation in performance among emergency departments (EDs),^34^ including between urban and rural coastal areas. We will characterise all UEC based on their type, location (eg, urban, rural coastal), the provision of other UEC services nearby (or in the same location) and deprivation of their catchment area.^35^ With the level of patient detail in the ECDS, we will develop bespoke metrics to capture some of the goals of innovative models in UECs in rural coastal areas. This will include measures to capture transfer avoidance and a proxy for the sensitivity and specificity of primary triage.

Analysis of variation will include linear regression models, using factors such as rurality, coastal location, major ethnic origin groups and socioeconomic deprivation (Index of Multiple Deprivation (IMD)quintiles) linked to ECDS through patients’ Lower Super Output Area (LSOA) of residence. This initial modelling approach will allow us to capture inequalities in performance and outcomes between and within individual EDs in rural coastal and non-rural coastal areas.

We will use information from WP1 on which innovative care models have been introduced and investigate their impacts on health and process outcome metrics developed in WP2.1. For example, we expect to analyse models such as SDEC, ambulatory assessment units, ARI hubs, hospital-at-home and virtual wards. Since these new UEC models may have different expected impacts on UEC performance, we will select the most relevant metrics to examine the impact of each innovative care model with input from our community groups and expert panel.

We will adopt a flexible approach to the analysis, with our study design tailored to the model of care under evaluation. Where we have clear start dates for new models of care within the time period of the dataset (WP1), we will use quasiexperimental approaches such as staggered difference in differences^36^ to assess the impact of the new emergency care models. These approaches for estimating causal effects will allow us to understand the impact of the new care models and explore variations across EDs that have implemented different models of emergency care.

Throughout our analysis, we will examine the implications of these innovative UEC models for health inequalities. We will present all results, summarising impacts by factors such as rurality, coastal location, major ethnic origin groups and socioeconomic deprivation quintiles (linked to ECDS through patients’ LSOA of residence).

#### Output

Alongside developing new UEC performance metrics, we will examine how performance varies between rural coastal and non-rural coastal areas using both new and standard measures. We will then assess the impact of new UEC models in rural coastal areas, providing evidence for regional and national discussions on responses to NHSE’s recovery plans for these communities. Stakeholders will be able to use our findings to identify providers with similar challenges that may benefit from adopting new models. These results will also guide the selection of case studies in WP3.

### WP 3: Case studies of purposively selected UEC models of care in rural coastal communities (months 13–27)

#### Aim

To provide a detailed understanding of which UEC models work for whom and in what context.

#### Design

We will select up to 12 models of innovative UEC provision in rural coastal areas as multi-method qualitative case sites. Case-site selection will be data-driven, using our WP 1 service map and performance metrics from WP 2.

Our case study design is deliberately flexible in the number of case sites required. This flexibility is essential to keep the project responsive to national policy changes (eg, new UEC models or priority areas) and to meet the needs of qualitative research at scale.^37^ From the experience of other national evaluations, certainty as to the number of sites required is not possible in the early stages of the project. We will initially estimate 12 case sites, but this number may be revisited.

Our case-site data collection will be undertaken in two iterations—using both ‘deep-dive’ (longer duration visits at a single site, up to 4 days) and ‘streamlined’ (shorter, more targeted visits to sites) visits—for both breadth and depth of data:

*Stage 1*. We will use data from WP 1 to create a long list of 10 potential case sites. We will then select three or four sites according to maximum variation criteria, across model type, geographic area, as well as other key site and contextual factors.
*Stage 2*. The sampling matrix will be updated based on WP 1’s service map and using new WP 2 performance data, as well as findings from case-site visits in Stage 1. Up to 20 potential sites will be considered, from which eight or nine streamlined case sites will be selected, and, using findings from WP 2, we will capture both ‘positive’ and ‘negative’ deviations from average performance. In this second round, we expect to focus less on collecting large amounts of data through in-depth visits and shorter, targeted visits. These will help us understand key differences between models (eg, workforce, performance) and explore aspects not covered in the first round.

We may also choose to break these up into repeat visits, with a number of streamlined visits over a month or two, to capture the impact of different demands and ‘compound pressures’ (eg, seasonal fluctuation) on services or experiences of different members of staff.

Data collection at case sites will include qualitative interviews with patients as well as staff working across UEC services (eg, emergency medicine, ambulance services, primary care, community health, social care, NHS 111, pharmacy) and non-participant observation of different aspects of the patient pathway and model (eg, team meetings and handovers between and within services, triage models). Depending on the case study data available, we will also seek to estimate the resource use of models. Data collection will begin with a familiarisation visit, followed by qualitative observations to gain an understanding of the case site and the key individuals and components of the model to interview and observe. Deep-dive visits will include a minimum of 12–15 hours of observation over a 3 or 4 day period at each site. Streamlined visits will occur over 1–2 days and will include brief 30 min observations of key aspects of the model and patient pathway.

Patients will be recruited based on a broad range of characteristics, following INCLUDE principles (eg, age, gender, ethnicity, reason for UEC attendance).^38^ Staff will be selected with maximum variation of professional group and band in mind (eg, doctors, nurses, allied health professionals, operational managers). Depending on the ownership of the UEC model and services involved, we will also interview ambulance personnel and/or staff from other local health and social services (eg, primary care, community health, NHS 111).

During interviews and observations, our focus will be on developing an in-depth understanding of how and why different UEC models influence patient clinical outcomes, experience, resource use and the workforce. Patient interviews will capture their care experience and general views on service configuration and accessing healthcare.

**Figure 2:**
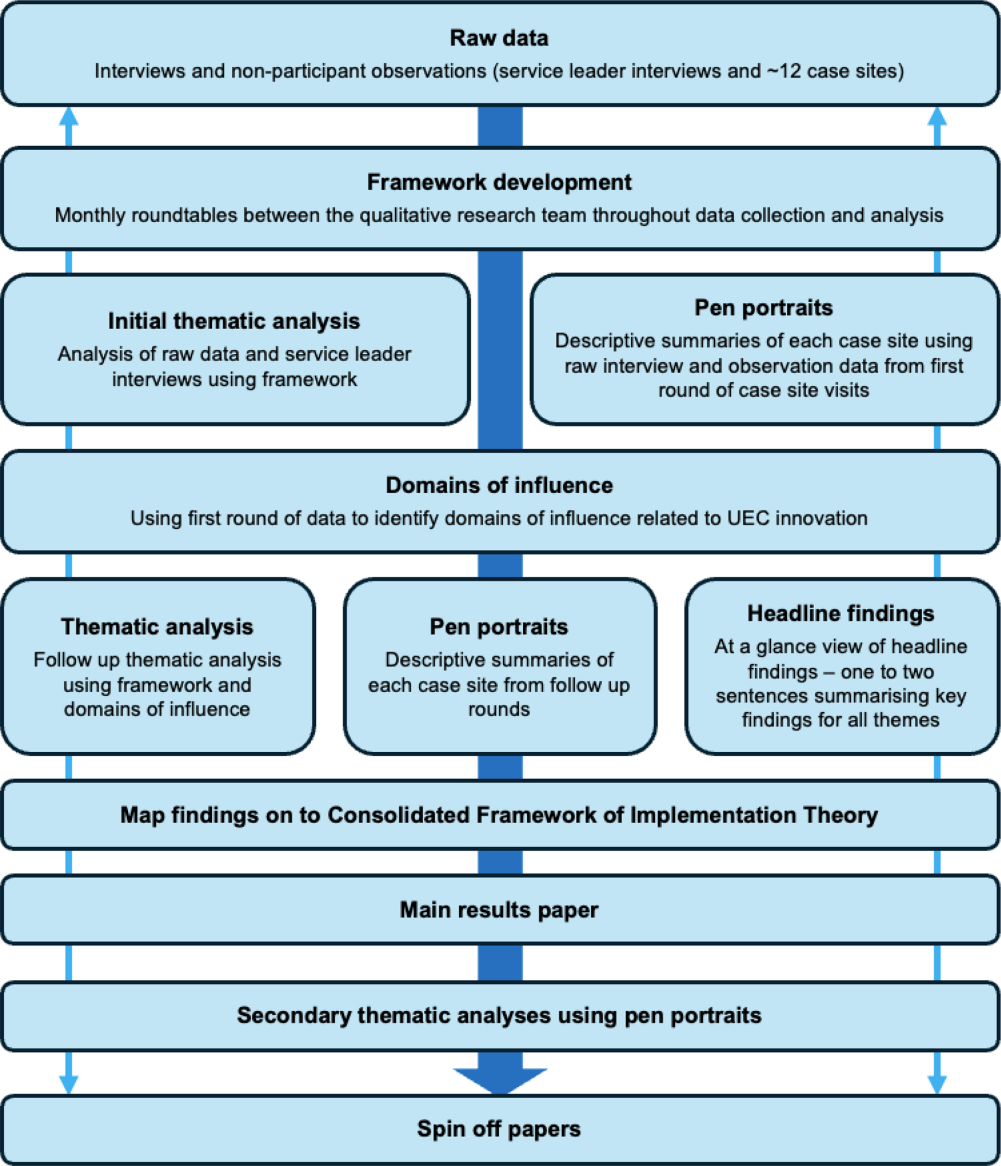
WP3 data analysis process. UEC, urgent and emergency care.

Data analysis will be based on our published approach to analysing large quantities of multi-method qualitative case-site data within national mixed-methods evaluations.^18 37^ Our interpretive approach utilises multiple analysis methods. The pen portrait method, as the focal point of analysis (see figure 2), will be fundamental to our approach as it will provide us with a mechanism for distilling large volumes of case-site data into case-site summaries (pen portraits), allowing detailed and contextualised analyses within and across case sites.^39^

### Mixed-methods integration

A core element of our analysis will involve using implementation theory to integrate and interpret qualitative and quantitative findings. Using the Consolidated Framework for Implementation Research (CFIR),^40^ we will identify aspects of UEC models that are promising and which have the potential to be scaled up and applied nationally. Following established approaches,^18 37 41^ data will be integrated in a mixed-methods matrix and mapped to CFIR domains to generate meta-inferences across sources.^42^ ‘Actionable findings’ will inform the identification of models for discussion at the end-of-project stakeholder event,^43^ with the aim of shaping future UEC provision and policy in rural coastal areas.

### Patient and public involvement

The project will take a community engagement and involvement (CEI) approach^44^ to patient and public involvement. This will remain central to Elevate throughout the study (see table 2). Our CEI approach was codeveloped in partnership with a community organisation in Scarborough—SeeChange. To achieve this, we cohosted dedicated events with residents who had recently attended Scarborough Hospital’s ED. Participants represented a range of ages (30–60 s), employment statuses, disabilities (including low vision, wheelchair use, and neurodevelopmental conditions), mental health conditions, chronic illnesses and caring roles.

**Table 2:** Community engagement and involvement across the study

Study timepoint	Description
Project development	Plans for involving patients and members of the public were designed alongside a rural coastal community in the North of England. We acted on advice from this group to take our research ‘into the community’ rather than having the community ‘come to us’.
WP1	CEI will be crucial in developing an appropriate and working definition of ‘rural coastal’ for our ‘service map’ and as the basis of subsequent work packages.
WP2	We will seek input from patients and members of the public to help us identify and interpret metrics for our analysis. For example, we will explore which UEC outcomes (eg, ED waiting times) are important to patients and why.
WP3	CEI contributors will be involved in developing study materials (eg, case-site topic guides for interviews) and selecting case sites. We will obtain advice on recruiting patients at case sites and identifying ways to ensure our case-site methodology is as inclusive as possible. We will engage with community groups while interpreting our study findings and identifying ways to facilitate feedback and dissemination to local communities.
Ad hoc	Regular engagement with a wide range of community networks and organisations will provide opportunities to receive input and advice on the project. For example, we will maximise the inclusivity of our research by addressing the priorities of a diverse range of people and giving voice to those who have historically not been represented or included within research. Engagement will also provide the chance for inclusive dissemination advice. For example, community engagement has shaped our EDI and dissemination plans. Feedback emphasised the prevalence of older adults, people with neurodiversity and those with long-term conditions in rural coastal areas. To improve accessibility, we will therefore use appropriate digital platforms for different audiences and ensure all materials are ‘easy read’ and visually accessible, with clear design and layout.

CEI, community engagement and involvement; ED, emergency department; EDI, equality, diversity and inclusion; UEC, urgent and emergency care.

Through close collaboration with SeeChange and the Scarborough community, we learnt that a bespoke community engagement approach would be needed. Rural coastal communities were described as ‘tight knit’ but often distrustful of ‘outsiders’ and the health and social care system. The representatives argued that people tended to remain within their own subcommunities and were reluctant to attend community engagement events held at the University or hospital settings. This feedback defined our approach to patient and public involvement and engagement (PPIE) within the Elevate study: as a single project-specific community advisory group would likely prove neither inclusive nor effective, we decided to ‘take our research into the community’, adopting a community-centred and community-designed model.

While CEI representatives will be included on the study steering committee and expert panel, most patient and public involvement will occur through the research team embedding within existing community groups to obtain feedback at key stages of the study. Through our CEI manager, we have access to a broad network of community groups across rural coastal North Yorkshire, enabling ongoing, bidirectional engagement and a constant feedback loop between the research team and community members. This approach enhances inclusivity and aligns with evidence on effective community engagement.^44^

This embedded engagement has directly shaped our approach to equality, diversity and inclusion (EDI) and dissemination. Community members highlighted the high prevalence of older people, individuals with neurodiversity and people living with multiple long-term conditions in rural coastal areas, prompting us to consider inclusion beyond ethnicity alone. To improve accessibility, community members recommended dissemination via platforms with reduced reliance on written content (eg, TikTok and Instagram) for people with neurodiversity and the ‘Nextdoor’ app for older people. Concerns were also raised that a solely written plain English summary could be exclusionary, particularly for those with neurodiversity or low literacy. In response, we will use easy-read formats for patient-facing materials wherever possible and apply inclusive design principles (eg, use of colour, font choice and text boxes). Together, these examples demonstrate how CEI feedback is embedded across study processes, including EDI and dissemination strategies.

## Ethics and dissemination

The Elevate study is registered through the Research Registry (researchregistry11126). Approval for qualitative components of WP1 and WP3 was obtained from the North of Scotland Research Ethics Committee (25/NS/0099). The University of Birmingham is the sponsor for the study. Informed consent will be taken from all participants across qualitative aspects of the project (WP1, WP3). Routine care is not altered by the study, and there are no significant ethical issues. WP2 will analyse routinely collected, pseudonymised national data (NHS ECDS and HES APC), accessed through a programme-level agreement between the Centre for Health Economics (University of York) and NHSE. This work therefore did not require NHS ethical approval. Approvals for further data sources (such as anonymised data from the ambulance service) will be obtained as required.

The study commenced in February 2025 and is expected to run for 30 months.

We have, in conjunction with patient and public partners and clinical advisors, developed a dissemination strategy that will ensure our study findings are disseminated in a way that makes them inclusive of and accessible to a wide range of stakeholders: academics (eg, journal publications and conferences), policymakers (eg, national conferences or national service meeting presentations and policy briefs) and patients (eg, accessible creative media, local TV and radio, and community engagement).
