# Supplementary material for: Identifying innovative models of urgent care in rural coastal areas in England: the Elevate study - a mixed-methods protocol
Source: BMJ Open. 2026 Feb 24;16(2):e116557. doi: 10.1136/bmjopen-2026-116557 (PMC12933752; doi:10.1136/bmjopen-2026-116557)
Supplement: Supplementary file 1 [file bmjopen-2026-116557.reviewer_comments.pdf]

PEER REVIEW HISTORY

BMJ Open publishes all reviews undertaken for accepted manuscripts. Reviewers are asked to complete a checklist review form and are provided with free text boxes to elaborate on their assessment. These free text comments are reproduced below.

ARTICLE DETAILS

Title (Provisional)

The Elevate study – identifying innovative models of urgent care in rural coastal areas in England: A mixed methods study protocol

Authors

Lampard, Pete; Adamson, Joy; Anderson, Helen; Ballantine, Lisa; Bell, Fiona; Benger, Jonathan Richard; Blakey, Rebecca Louise; Dickinson, Phillip; Dykes, Steven; Gaughan, James; Maitland-Knibb, Stuart; Mensah, Daniel; Ransome, Zacharia Alice; Richardson, Gerry; Santos, Rita; Sheridan, Rebecca; Sivey, Peter; Smith, Ed; Song, Wei; Sutcliffe, Martin; Stockwell, Cameron Jack; Voss, Sarah; Scantlebury, Arabella

---

This article was not externally reviewed at BMJ Open. Protocols that have been independently assessed prior to submission to BMJ Open are usually fast-tracked to publication on the grounds that further substantial changes will not be possible. This independent assessment will usually be external, independent review for both a substantial grant award from a non-commercial or government funder and ethics approval.
